# Public vs Private: Comparing resource availability and practitioner preferences in treating individuals diagnosed with Personality Disorders in Iași, Romania

**DOI:** 10.1192/j.eurpsy.2026.11468

**Published:** 2026-07-31

**Authors:** M. Hugianu-Cozma, R.-D. Hugianu, P. R. Dobrin

**Affiliations:** 1 IX Cronici, Institutul de Psihiatrie Socola; 2 Universitatea de Medicina si Farmacie “Grigore T. Popa”, Iasi, Romania

## Abstract

**Introduction:**

Personality disorder (PD) is defined as an enduring, pervasive, and inflexible pattern of inner experience and behaviour that deviates markedly from the expectations of an individual’s culture, leading to significant distress or impairment. (DSM-5) The first-line treatment for PD is psychotherapy, but medication may be used to manage co-occurring psychiatric symptoms.

**Objectives:**

We aim to explore the differences in the availability of resources for treating individuals with PDs between the Public (Pub) and the Private (Pri) sectors of mental health services in Iași, and how this is influencing the practitioners in supporting this vulnerable category. We also wanted to gain more clarity on the practitioners’ overall strategies of addressing this, and their subjective views on the shortcomings.

**Methods:**

The study is designed as an exploratory one, utilising a 16-item survey that we created specifically for this study. Questions are addressed in a closed manner, giving the respondents items to select from, and 2 of them have quantitative answers. Inclusion criteria consist of: being a fully trained psychiatrist, practicing in Iași – Romania, and acceptance of informed consent. Surveys were anonymous and distributed in person by the authors. The answers were centralised and analysed through descriptive statistics.

**Results:**

There was a total of 49 respondents, of whom 29 are working in the public sector. The most frequent PD cluster was B (82.75% Pub, 70% Pri). All respondents have access to medication; Psychotherapy is available for 27.58% Pub and 75% Pri; Counselling 65.51% Pub and 85% Pri; Occupational therapy 17.24% Pub and 5% Pri; Social worker for 13.79% Pub and 0 Pri; No therapeutic groups in Pub and 30% Pri. All respondents in Pub are using medication as first line, compared to 50% in Pri; 24.13% in Pub consider psychotherapy as first line compared to 60% for Pri. Average waiting time for psychotherapy, including if collaborating with someone outside their service, was 2.37 weeks Pub (11 respondents) 1.88 weeks Pri (17 respondents). Only 24.13% of respondents were satisfied with the availability of resources in their practice for Pub, and 85% for Pri.

**Image 1:**
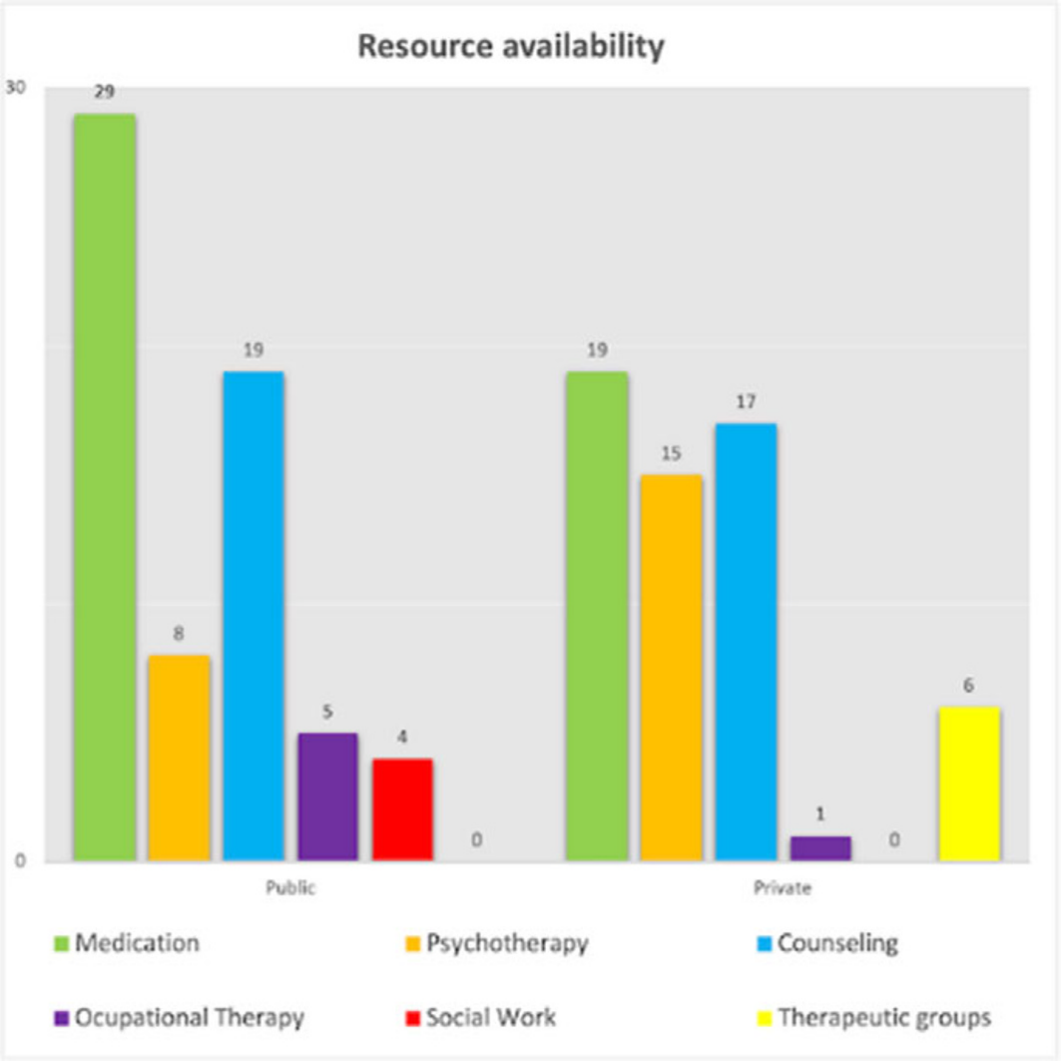
Image 1: Long description.

**Image 2:**
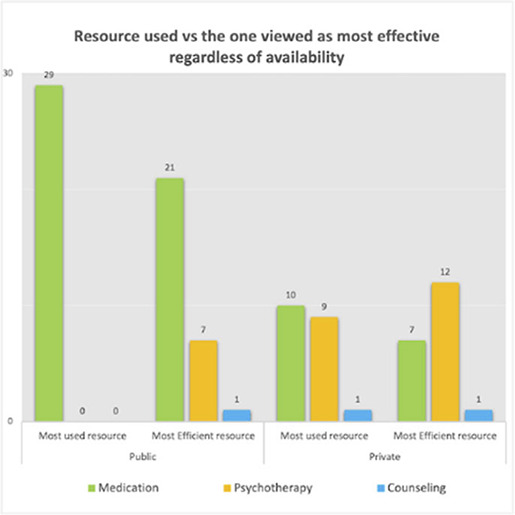
Image 2: Long description.

**Conclusions:**

The results above highlight a major discrepancy in what practitioners have available for supporting individuals diagnosed with PDs, in relation to the sector they work in. The private sector seems to adapt better to treatment guidelines and try to overcome the shortcomings of the public sector. Additionally, results suggest that the lack of resources has an impact on how practitioners recommend certain treatments, highlighted by the fact that the vast majority of psychiatrists in the public sector view medication as the most efficient in treating individuals with PDs.

**Disclosure of Interest:**

None Declared

## Long descriptions

### Image 1: Long description

The vertical Y axis represents frequency from 0 to 30. The horizontal X axis categorizes data into Public and Private sectors. A legend at the bottom identifies six color-coded resources.

In the Public sector, from left to right

- Medication (green) has a value of 29.

- Psychotherapy (orange) has a value of 8.

- Counseling (light blue) has a value of 19.

- Occupational Therapy (purple) has a value of 5.

- Social Work (red) has a value of 4.

- Therapeutic groups (yellow) has a value of 0.

In the Private sector, from left to right

- Medication (green) has a value of 19.

- Psychotherapy (orange) has a value of 15.

- Counseling (light blue) has a value of 17.

- Occupational Therapy (purple) has a value of 1.

- Social Work (red) has a value of 0.

- Therapeutic groups (yellow) has a value of 6.

Navigate back to Image 1:.

### Image 2: Long description

A bar chart titled Resource used vs the one viewed as most effective regardless of availability. The y-axis represents a numerical scale from 0 to 30. The x-axis is divided into two main categories: Public and Private. Each category is further split into Most used resource and Most Efficient resource. A legend at the bottom identifies three resources: Medication (green), Psychotherapy (yellow), and Counseling (blue).

In the Public sector:

* Most used resource: Medication is at 29, while Psychotherapy and Counseling are both at 0.

* Most Efficient resource: Medication is at 21, Psychotherapy is at 7, and Counseling is at 1.

In the Private sector:

* Most used resource: Medication is at 10, Psychotherapy is at 9, and Counseling is at 1.

* Most Efficient resource: Medication is at 7, Psychotherapy is at 12, and Counseling is at 1.

Navigate back to Image 2:.

